# Validation of non-invasive indicators in the screening of metabolic dysfunction-associated fatty liver disease: a cross-sectional study among Uighurs in rural Xinjiang

**DOI:** 10.1186/s40001-023-01536-2

**Published:** 2023-12-02

**Authors:** Yanbo Guo, Yunhua Hu, Jing Yang, Rulin Ma, Xianghui Zhang, Heng Guo, Xinping Wang, Yu Li, Xinyu Peng, Shijie Zhang, Jia He, Shuxia Guo

**Affiliations:** 1 Department of Public Health, Shihezi University School of Medicine, Suite 721, Beier Road, Shihezi, 832000 China; 2 Department of Hepatobiliary Surgery, The First Affiliated Hospital of Shihezi University School of Medicine, Shihezi, 832000 China; 3 Department of NHC Key Laboratory of Prevention and Treatment of Central, Asia High Incidence Diseases, The First Affiliated Hospital of Shihezi University School of Medicine, Shihezi, 832000 China

**Keywords:** Metabolic dysfunction-associated fatty liver disease, Steatosis index, Non-invasive indicators, Screening value

## Abstract

**Background:**

Metabolic dysfunction-associated fatty liver disease (MAFLD) is the most common chronic liver disease in China. Our study aimed to evaluate the screening value of the fatty liver index (FLI), hepatic steatosis index (HSI), lipid accumulation product (LAP), visceral adiposity index (VAI), and Zhejiang University index (ZJU), as well as other single indicators for MAFLD. We aimed to find the optimal screening tool and its appropriate cut-off values for rural Uyghur adults.

**Methods:**

We completed a survey of 14,321 Uyghur adults in 51 groups in Kashgar, Xinjiang, in 2016 using a typical sampling method, with 12,794 patients ultimately included in statistical analyses. Fatty liver was diagnosed using ultrasonography.

**Results:**

The prevalence of fatty liver disease (FLD) and MAFLD was 16.73% and 16.55%, respectively, and the FLI, HSI, LAP, VAI, and ZJU were all independently associated with an increased risk of MAFLD. The areas under the receiver operating characteristic curves (AUCs) of the FLI for diagnosing MAFLD in men and women were 0.853 and 0.847, respectively. The AUCs of the body mass index (BMI) for diagnosing MAFLD in men and women were 0.850 and 0.852, respectively. Compared with other metabolic-related markers, FLI had the largest AUC. In men, the optimal cut-off values of FLI and BMI for diagnosing MAFLD were 45 (sensitivity 84.83%, specificity 69.57%) and 27.4 (sensitivity 78.47%, specificity 76.30%), respectively. In women, the optimal cut-off values of FLI and BMI for diagnosing MAFLD were 45 (sensitivity 80.11%, specificity 74.23%) and 28.0 (sensitivity 79.56%, specificity 75.41%), respectively. In men and women, an FLI score of < 30 ruled out MAFLD, while a score of ≥ 50 was a basis for diagnosis.

**Conclusion:**

FLI and BMI had good screening ability for MAFLD and were superior to HSI, LAP, VAI, and ZJU in both sexes.

## Introduction

In 2020, non-alcoholic fatty liver disease (NAFLD) was renamed metabolic-associated fatty liver disease (MAFLD) [1]. MAFLD is a chronic disease characterized by hepatic fat accumulation and underlying metabolic dysregulation. The condition is a significant public health issue, affecting more than a quarter of adult populations worldwide [2]. Studies have shown that MAFLD induces hepatic lipotoxicity and mitochondrial dysfunction leading to hepatocyte apoptosis, inflammation, and fibrosis [3]. In addition to its close association with liver disease, MAFLD also plays an important role in the progression of various extrahepatic diseases, including cardiovascular disease, diabetes, and chronic kidney disease [4]. In China, the disease burden of MAFLD has surpassed that of other chronic liver diseases [5, 6]. However, the current diagnosis and treatment rates of MAFLD are low, and interventions geared to its harmful effects may be insufficient [7]. Therefore, to optimize the management of patients with MAFLD, simple and effective early screening tools are necessary.

Among the non-invasive indicators currently used for NAFLD screening, the fatty liver index (FLI), hepatic steatosis index (HSI), lipid accumulation product (LAP), visceral adiposity index (VAI) and Zhejiang University index (ZJU) are widely used and have been validated in different populations [8–10]. However, the screening value of these indices may need further verification under the new definition of MAFLD. The above indexes are established based on routine measurements in clinical practice, and compared with liver biopsy and ultrasonography, may reduce medical burdens. However, differences in the cut-off values in different populations and regions exist, and the cut-off values in previous studies may not apply to the Uyghur population in rural Xinjiang. These values may require adjustments through external verification prior to practical application.

Therefore, this study aimed to evaluate the accuracy of non-invasive indicators in diagnosing MAFLD in rural Uyghur populations in Xinjiang as well as determine their optimal cut-off values and ranges.

## Materials and methods

### Study population

This cross-sectional study was conducted in Kashgar, Xinjiang, from August to September 2016. Our research is based on the 51st Regiment of the Farm, which is the only regiment with Uyghurs as its main inhabitants. Using the typical sampling method, the sixth, eighth, 11th, 12th, and 13th companies of the 51st Regiment of the Farm were selected as the survey sites. The sixth, eighth, 11th, 12th, and 13th companies are one level below the farm. If farms are like towns, companies are like villages. We chose these companies with a large number of Uyghurs to carry out the investigation and research in a concentrated way. A total of 14,321 permanent Uyghur residents aged ≥ 18 years were interviewed after obtaining informed consent. We excluded a total of 1527 participants, including long-term migrants, pregnant women, those who could not cooperate with the investigation (*n* = 317), participants with incomplete basic information (*n* = 971), and participants with missing ultrasound information (*n* = 239). Ultimately, 12,794 participants were included in statistical analyses.

### Data collection

The questionnaire survey and physical examination were conducted by uniformly trained investigators through on-site centralized interviews. The questionnaire included general demographic information, medical history, and family history. Physical examinations included height, weight, body mass index (BMI), waist circumference (WC), hip circumference (HC), systolic blood pressure (SBP), and diastolic blood pressure (DBP). Before the measurement, participants were required to remove excess clothing, hats, and shoes. Waist circumference was measured with an inelastic band at the midpoint of the line between the anterior superior iliac crest and the inferior margin of the 12th costal. Hip circumference was measured around the hip through the most prominent part of the gluteus maximus. Blood pressure was measured after participants had rested in a sitting position for 5 min. Each measurement required an interval of one minute, and the mean value after three measurements was used. These measurement methods were consistent with existing research among this group [11]. Type 2 diabetes mellitus (T2DM) was diagnosed according to the American Diabetes Association 2012 criteria [12].

Venous blood samples were collected from all participants after overnight fasting. All parameters, including fasting plasma glucose (FPG), triglycerides (TG), total cholesterol (TC), high-density lipoprotein cholesterol (HDL-C), low-density lipoprotein cholesterol (LDL-C), aspartate aminotransferase (AST), alanine aminotransferase (ALT), and gamma-glutamyl transferase (GGT), were measured using an automatic biochemical analyzer (Olympus Diagnostics, Hamburg, Germany) at our laboratory department.

The calculation formula used in this study was as follows:

$$\mathrm{FLI}=\frac{{\mathrm{e}}^{0.953\times \mathrm{loge }\left (\mathrm{TG}\right)+0.139\times \mathrm{BMI}+0.718\times \mathrm{loge } (\mathrm{GGT})+0.053\times \mathrm{WC }-15.745}}{1+{\mathrm{e}}^{0.953\times \mathrm{loge }\left (\mathrm{TG}\right)+0.139\times \mathrm{BMI}+0.718\times \mathrm{loge } (\mathrm{GGT})+0.053\times \mathrm{WC}-15.745} }\times 100$$ [13];

HIS = 8× (ALT/AST ratio)+BMI (+2,if women; +2,if diabetes mellitus) [14];

LAP for men = (WC [cm] - 65) × (TG[mmol/L]); LAP for women = (WC [cm] - 58) × (TG [mmol/L]) [15] 

ZJU = BMI (kg/m^2^) + FPG (mmol/L) + TG (mmol/L) + (3 × ALT/AST Ratio) (+2, If Women) [16] 

VAI = (WC/ (39.68 + (1.88 × BMI)))× (TG/1.03) × (1.31/HDL-C) for Men; (WC/ (36.58 + (1.89 × BMI))) × (TG/0.81) × (1.52/HDL-C) for Women [17] 

### Definition of MAFLD

MAFLD [18] was defined as the presence of one of the following three criteria based on hepatic steatosis: (1) overweight/obesity (BMI ≥ 23 kg/m^2^); (2) presence of T2DM (FPG ≥ 7.1 mmol/L); (3) lean/normal weight (BMI < 23 kg/m^2^) with metabolic dysregulation. Metabolic dysregulation was defined as the presence of at least two of the following metabolic risk abnormalities: (a) WC ≥ 90/80 cm in men and women, respectively; (b) blood pressure ≥ 130/85 mmHg; (c) TG ≥ 1.70 mmol/L; (d) HDL-C < 1.0 mmol/L in men and < 1.3 mmol/L in women; (e) Prediabetes: FPG level of 5.6–6.9 mmol/L. Hepatic steatosis was determined by ultrasound examination performed by a specialist clinician.

## Statistical analyses

Categorical and continuous variables were expressed as frequency (percentage) and mean ± standard deviation, respectively, and the Chi-square test and Mann–Whitney *U* test were used for comparison between groups. Factors influencing MAFLD were determined using univariate analyses. Except for the MAFLD components, the remaining influencing factors were included in the multivariate analyses. Logistic regression models were constructed using the step-forward method to estimate the odds ratios (ORs) and 95% confidence intervals (CIs) for FLI, HSI, and MAFLD. The final model was adjusted for age, sex, and HC, TC, LDL-C, AST, ALT, and GGT levels. Areas under the receiver operating characteristic (ROC) curve (AUC), sensitivity, specificity, positive likelihood ratio (LR_+_), negative likelihood ratio (LR_-_), positive predictive value (PPV), negative predictive value (NPV), and Youden index were used to evaluate the screening ability of non-invasive markers for MAFLD. All analyses were performed using two-sided tests, with *p* < 0.05 considered statistically significant. The Delong test was used to compare whether the AUC differences of the different receiver operating characteristic (ROC) curves were statistically significant. All statistical analyses were performed using SPSS 26.0 and R 4.1.1.

## Results

### Basic demographic characteristics

Among the 12,794 Uyghur adults included, the mean age was 38.10 ± 14.09 years, and 6448 (50.40%) men participated. Of these patients, 2141 (16.73%) had fatty liver disease (FLD) and 2118 (16.55%) had MAFLD. Compared with the non-MAFLD group, the MAFLD group was older; had higher BMI, WC, HC, SBP, DBP, TG, TC, LDL-C, AST, ALT, GGT, FPG, FLI, HSI, LAP, VAI, and ZJU levels; included more female patients; had a higher prevalence of T2DM (all *p* < 0.001); and had lower HDL-C levels (*p* < 0.001) (Table 1).

**Table 1:** Basic demographic characteristics grouped by the presence of metabolic dysfunction-associated fatty liver disease

Variable	Overall	MAFLD		*p*
		Yes	No	
n	12,794	2118 (16.55)	10,676 (83.45)	
Age (years)	38.10 ± 14.09	44.86 ± 12.06	36.76 ± 14.08	< 0.001
Sex				0.031
Men	6448 (50.40)	1022 (48.25)	5426 (50.82)	
Women	6346 (49.60)	1096 (51.75)	5250 (49.18)	
BMI (kg/m^2^)	25.77 ± 4.77	31.37 ± 5.00	25.04 ± 4.14	< 0.001
WC (cm)	90.88 ± 13.47	102.54 ± 12.70	88.56 ± 12.37	< 0.001
HC (cm)	100.83 ± 7.92	106.82 ± 8.70	99.64 ± 7.18	< 0.001
SBP (mm Hg)	128.91 ± 20.27	138.07 ± 22.23	127.09 ± 19.34	< 0.001
DBP (mm Hg)	75.21 ± 15.20	80.00 ± 13.46	74.26 ± 15.34	< 0.001
TG (mmol/L)	1.72 ± 1.46	2.39 ± 1.90	1.59 ± 1.32	< 0.001
TC (mmol/L)	4.75 ± 2.04	5.27 ± 2.20	4.65 ± 1.99	< 0.001
HDL-C (mmol/L)	1.57 ± 0.56	1.53 ± 0.60	1.57 ± 0.55	< 0.001
LDL-C (mmol/L)	2.65 ± 0.83	2.86 ± 1.02	2.61 ± 0.78	< 0.001
AST (U/L)	30.26 ± 24.14	36.86 ± 27.02	28.95 ± 23.31	< 0.001
ALT (U/L)	24.54 ± 13.35	25.72 ± 12.38	24.32 ± 13.52	< 0.001
GGT (U/L)	19.74 ± 17.33	26.36 ± 21.58	18.04 ± 15.61	< 0.001
FPG (mmol/L)	4.99 ± 2.04	5.62 ± 2.99	4.86 ± 1.77	< 0.001
T2DM	697 (5.4)	260 (12.3)	437 (4.1)	< 0.001
FLI	38.15 ± 28.27	68.94 ± 23.71	32.04 ± 24.92	< 0.001
HSI	35.58 ± 15.79	40.02 ± 15.21	34.70 ± 15.75	< 0.001
LAP	61.48 ± 65.60	109.09 ± 93.86	52.04 ± 53.58	< 0.001
VAI	2.02 ± 2.21	2.90 ± 2.85	1.85 ± 1.99	< 0.001
ZJU	36.94 ± 8.26	43.17 ± 8.36	35.71 ± 7.66	< 0.001

Values are presented as means ± standard or *n* (%)

*MAFLD* metabolic dysfunction-associated fatty liver disease, *BMI* body mass index, *WC* waist circumference, *HC* hip circumference, *SBP* systolic blood pressure, *DBP* diastolic blood pressure, *TG* triglycerides, *TC* total cholesterol, *HDL-C* high-density lipoprotein cholesterol, *LDL-C* low-density lipoprotein cholesterol, *AST* aspartate aminotransferase, *ALT* alanine aminotransferase, *GGT* gamma-glutamyl transferase, *FPG* fasting plasma glucose, *SCr* serum creatinine, *eGFR* estimated glomerular filtration rate, *T2DM* type 2 diabetes mellitus, *FLI* fatty liver index, *BMI* body mass index, *ZJU* Zhejiang University Index, *WC* waist circumference, *LAP* lipid accumulation product, *HSI* hepatic steatosis index, *VAI* visceral adiposity index

### Relationship between non-invasive indicators and MAFLD

In the interaction analysis, we considered the indicators that were not involved in the definition of liver steatosis indexes and MAFLD: age, hip circumference, total cholesterol, and sex. The results showed that the OR value was 1 for all indicators except sex. Further analyses were stratified by sex due to the interaction between steatosis indexes (FLI, HSI, LAP, VAI and ZJU) and sex on the prevalence of MAFLD (*p* < 0.001). After multivariate adjustment, FLI, HSI, BMI, TG, LDL-C, HC, FPG, WC, TC, AST, GGT, LAP, VAI, ZJU, ALT, and HDL-C variables remained significantly associated with MAFLD in men, while the correlation between ALT, HDL-C, and MAFLD was not statistically significant in women (*p* > 0.05). In both men and women, associations with LDL-C, ZJU, HC, and MAFLD were stronger compared with other variables, with the exception of the MAFLD components of BMI and TG (Table 2).

**Table 2:** ROC analyses and logistic regression analyses of variables and metabolic dysfunction-associated fatty liver disease

Variable	Men			Women		
	AUC (95%CI)	OR (95%CI)	*p*	AUC (95%CI)	OR (95%CI)	*p*
FLI	0.853 (0.841–0.865)	1.045 (1.043–1.048)	< 0.001	0.847 (0.835–0.859)	1.050 (1.047–1.053)	< 0.001
BMI (kg/m^2^)	0.850 (0.838–0.862)	1.315 (1.285–1.345)	< 0.001	0.852 (0.840–0.864)	1.261 (1.236–1.287)	< 0.001
ZJU	0.834 (0.821–0.846)	1.116 (1.101–1.131)	< 0.001	0.842 (0.830–0.853)	1.105 (1.091–1.120)	< 0.001
WC (cm)	0.803 (0.789–0.817)	1.075 (1.066–1.083)	< 0.001	0.800 (0.786–0.814)	1.052 (1.044–1.060)	< 0.001
LAP	0.779 (0.764–0.794)	1.007 (1.006–1.008)	< 0.001	0.761 (0.746–0.776)	1.005 (1.004–1.007)	< 0.001
HSI	0.745 (0.729–0.761)	1.017 (1.009–1.025)	< 0.001	0.772 (0.758–0.787)	1.035 (1.026–1.043)	< 0.001
HC (cm)	0.738 (0.722–0.755)	1.109 (1.097–1.121)	< 0.001	0.771 (0.755–0.786)	1.117 (1.106–1.128)	< 0.001
TG (mmol/L)	0.704 (0.686–0.721)	1.241 (1.187–1.298)	< 0.001	0.664 (0.647–0.681)	1.143 (1.085–1.203)	< 0.001
VAI	0.698 (0.681–0.716)	1.192 (1.148–1.238)	< 0.001	0.659 (0.642–0.676)	1.045 (1.017–1.075)	0.002
AST (U/L)	0.668 (0.649–0.686)	1.011 (1.008–1.015)	< 0.001	0.619 (0.600–0.637)	1.008 (1.004–1.011)	< 0.001
TC (mmol/L)	0.652 (0.634–0.670)	1.101 (1.062–1.14)	< 0.001	0.644 (0.627–0.662)	1.046 (1.017–1.075)	0.002
GGT (U/L)	0.650 (0.630–0.670)	1.020 (1.015–1.024)	< 0.001	0.593 (0.573–0.612)	1.015 (1.009–1.02)	< 0.001
FPG (mmol/L)	0.606 (0.587–0.626)	1.086 (1.055–1.119)	< 0.001	0.595 (0.576–0.614)	1.065 (1.031–1.101)	< 0.001
LDL-C (mmol/L)	0.582 (0.562–0.602)	1.168 (1.066–1.281)	0.001	0.596 (0.577–0.615)	1.269 (1.157–1.392)	< 0.001
ALT (U/L)	0.568 (0.548–0.588)	0.992 (0.985–0.998)	0.013	0.546 (0.527–0.565)	0.997 (0.989–1.004)	0.387
HDL-C (mmol/L)	0.448 (0.427–0.468)	0.799 (0.692–0.922)	0.002	0.465 (0.445–0.485)	0.944 (0.831–1.073)	0.379

All models were adjusted for sex, age, HC, TC, LDL-C, AST, ALT, and GGT levels

### Screening value of non-invasive indicators for MAFLD

**Fig. 1:**
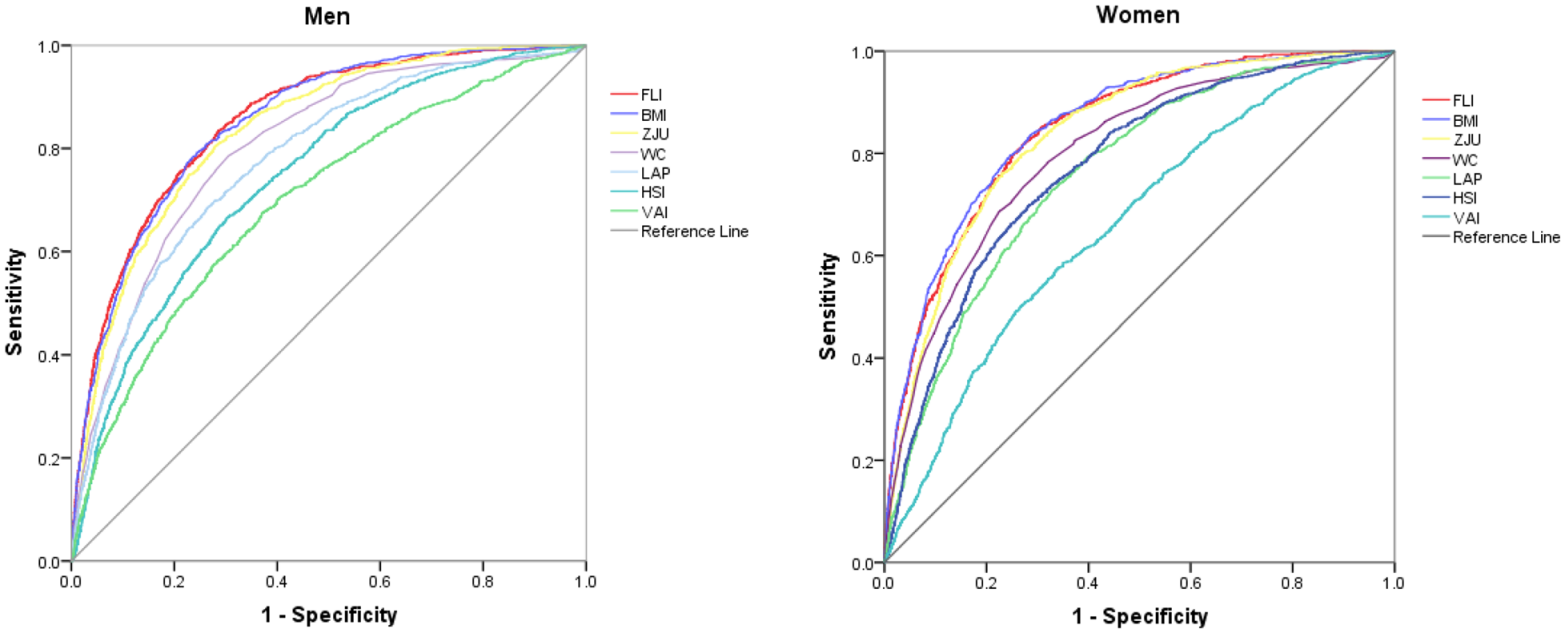
ROC curves for FLI, BMI, ZJU, WC, LAP, HSI, and VAI in predicting the occurrence of metabolic dysfunction-associated fatty liver disease in men and women. (*ROC* receiver operating characteristic, *FLI* fatty liver index, *BMI* body mass index, *ZJU* Zhejiang University Index, *WC* waist circumference, *LAP* lipid accumulation product, *HSI* hepatic steatosis index, *VAI* visceral adiposity index, *MAFLD* metabolic dysfunction-associated fatty liver)

ROC curve analyses showed that FLI had the strongest screening ability for MAFLD in men, with an AUC greater than those of BMI, ZJU, WC, LAP, HSI, HC, TG, VAI, AST, TC, GGT, FPG, ALT, HDL-C, and LDL-C. In women, the FLI was second only to BMI in terms of AUC for diagnosing MAFLD, but the AUC was not statistically different (*Z* = 1.039, *p* = 0.30 > 0.05) (Fig. 1). AUCs for the FLI and BMI were 0.853 (0.841–0.865) and 0.850 (0.838–0.862) in men, and 0.847 (0.835–0.859) and 0.852 (0.840–0.864) in women, respectively (Table 2).

### Selection of optimal critical value and diagnostic range of FLI and BMI for diagnosing MAFLD

Table 3 shows the sensitivity and specificity of cut-off points at 5-point intervals for the FLI. The optimal critical value was determined using the maximum Youden index method. The optimal cut-off values of FLI and BMI for diagnosing MAFLD in men were 45 (sensitivity, 84.83%; specificity, 69.57%) and 27.4 (sensitivity, 78.47%; specificity, 76.30%), respectively. Additionally, the optimal cut-off values for FLI and BMI in women were 45 (sensitivity, 80.11%; specificity, 74.23%) and 28.0 (sensitivity, 79.56%; specificity, 75.41%), respectively.

**Table 3:** Selection of optimal cut-off values and diagnostic ranges for FLI and BMI

Index	Cut-off value	Sensitivity (%)	Specificity (%)	LR_+_	LR_-_	PPV (%)	NPV (%)	Youden
FLI								
Men	30	94.03	53.21	2.01	0.11	27.44	97.93	0.465
	45	84.83	69.57	2.79	0.22	34.43	96.06	0.544
	50	79.94	74.05	3.08	0.27	36.72	95.15	0.540
Women	30	89.51	60.30	2.25	0.17	32.02	96.52	0.498
	45	80.11	74.23	3.11	0.27	39.35	94.70	0.543
	50	75.09	77.87	3.39	0.32	41.44	93.74	0.530
BMI								
Men	27.4	78.47	76.30	3.31	0.28	37.95	94.94	0.548
Women	28.0	79.56	75.41	3.23	0.27	40.31	94.64	0.550

*FLI* fatty liver index, *BMI* body mass index, *LR+* positive likelihood ratio, *LR-* negative likelihood ratio, *PPV* positive predictive value, *NPV* negative predictive value

The sensitivity and specificity did not reach 100% at the optimal cut-off value, indicating an area of overlap between patients and non-patients. Therefore, we determined the diagnostic range of the FLI according to the method described by Chen et al. [19], where an FLI < 30 (men, LR_−_ = 0.13; women, LR_−_ = 0.18) ruled out MAFLD, while an FLI ≥ 50 (men, LR_+_  = 3.05; women, LR_+_  = 3.37) was a basis for its diagnosis (Table 3).

## Discussion

In this cross-sectional study conducted among 12,794 Uyghur adults in rural Xinjiang, we assessed the association and screening ability of five steatosis indexes (FLI, HSI, LAP, VAI, ZJU) for MAFLD. We also compared them with traditional indicators, including BMI, WC, LAP, HC, TC, AST, GGT, and LDL-C. The results indicated that higher steatosis index levels were positively associated with an increased risk of MAFLD. The FLI, HSI, LAP, and ZJU have good screening values for MAFLD in both men and women, among which the FLI had the best screening ability.

MAFLD is the hepatic manifestation of metabolic syndrome, which is complex and has many risk factors. A recent study showed that MAFLD tended to coexist with systemic metabolic disorders [20]. Similarly, our study found that MAFLD was affected by various metabolism-related factors, such as WC, HC, blood lipid level, AST, ALT, GGT, LAP, and T2DM. The risk of developing MAFLD has also been reported to vary widely according to race and geographic area [21]. Preliminary studies have found that compared with other ethnic groups, Uyghur residents eat fewer vegetables and have a higher intake of staple foods, red meat, dairy products, vegetable oil, and salt [22, 23]. Although the study population was from a rural area with a backward economy, the BMI and WC were significantly higher than those of the Chinese average [24, 25], and the prevalence of MAFLD was lower than that of the Chinese average [21]. This may be related to the fact that Uyghurs have a fat distribution that is beneficial to metabolic health [26, 27], the average age of the study population is lower, and most of them are engaged in physical labor-related occupations.

In this study, after adjusting for age, sex, HC, TC, LDL-C, AST, ALT, and GGT, high FLI, HSI, LAP, VAI, and ZJU scores remained significantly associated with MAFLD. The FLI is independently associated with an increased risk of hypertension, cardiometabolic disease, atherosclerosis, T2DM, and worsening renal function [28–31]. Moreover, HSI has been associated with carotid atherosclerosis and chronic kidney disease [32, 33]. Both the LAP and VAI have been strongly associated with metabolic syndrome, T2DM, and cardiovascular events [17, 34, 35], and the ZJU has been associated with insulin resistance [36]. These findings suggest that the five steatosis indexes may be used for both MAFLD screening as well as identifying high-risk metabolic-related and cardiovascular diseases groups. Therefore, patients with high steatosis indexes should undergo more precise clinical examinations. Additionally, conducting early lifestyle interventions, and preventing cardiovascular disease are vital.

Among the existing non-invasive evaluation indicators of hepatic steatosis, the FLI is the most widely used and has been recommended by international guidelines to be used in large epidemiological studies [1], followed by HSI and ZJU. The LAP and VAI, as new body fat indicators, have also attracted increasing attention. The FLI was developed by Bedogni et al. [13] in an Italian population with a mean age of 58 years, and the AUC for the diagnosis of FLD by ultrasound was 0.84 (95% CI 0.81–0.87). This study showed that FLI scores of < 30 and ≥ 60 were cut-offs for the presence or absence of FLD, respectively. At present, the ability of the FLI to screen for FLD or NAFLD has been validated in some populations, with AUCs of 0.813 for White and elderly populations [37]; 0.827 for Taiwanese populations [38]; and 0.834 for middle-aged and elderly populations in Shanghai [39]. Similarly, our study found that the FLI had the best screening power compared with the other single indicators and that FLI appeared to perform better in the diagnosis of MAFLD compared with that of FLD or NAFLD. However, not all studies support that FLI is optimal for its diagnostic value. For example, Kim et al. [40] showed that the FLI had an AUC of 0.785 in Korean populations, and that its diagnostic value was weaker than that of BMI and WC. This may have been due to lower BMI and WC levels and higher TG and GGT levels in the Korean populations compared with Italian populations. Our study showed that the screening power of BMI for MAFLD was comparable to that of FLI in women, possibly due to BMI being an essential component of MAFLD. These results also strongly suggest that being overweight or obese is the primary cause of the current MAFLD epidemic [20, 41]. The original study of the HSI and ZJU showed good accuracy in predicting NAFLD. Kenichiro et al. [42] found that the AUCs of the HSI and ZJU in Japanese health examiners were 0.874 and 0.886, respectively. Duan et al. [8] showed that the AUCs of LAP and VAI in different sex and body weight subgroups was > 0.7. Compared with these findings, the AUCs of the ZJU and VAI in this study were slightly lower, which may be due to the differences in the included population and diagnostic criteria of diseases. We also found that the diagnostic value of the FLI was significantly better than that of the HSI, LAP, VAI, and ZJU, consistent with reports from related studies [43]. Although the results of this study suggest that the diagnostic value of FLI was slightly higher than that of BMI, considering that BMI does not require any blood tests, it may be more convenient and practical as a simple screening tool for MAFLD in the general population.

This study had high sensitivity and specificity at the optimal cut-off value of the FLI for screening MAFLD. However, the low PPV and high NPV of the FLI and HSI may have been due to the low prevalence of MAFLD in this study population. Moreover, compared with the study by Duan et al. [8], the sensitivity and specificity at the optimal cut-off value of BMI in this study were higher, which may be related to the differences in the population and sample sizes.

Currently, approximately 36.11% of China's population lives in rural areas [44]. Simplifying the diagnosis of MAFLD could help achieve health equity and close gaps in the quality of primary health care [5]. No specific treatment for MAFLD exists [45]; however, the risk of MAFLD can be significantly reduced by reducing BMI and WC, which may be accomplished through furthering public knowledge and dietary interventions. In addition, early screening for MAFLD in young populations may prevent further progression of the disease, which may in turn reduce the burden of disease involved with a declining workforce and loss of social value. Therefore, validation of the simple screening tools for MAFLD in this study may have important implications for clinical practice and the development of primary health care services.

## Strengths and limitations

This study was the first to evaluate the screening value of the FLI and HSI for MAFLD in large-scale Uyghur populations. However, there were some limitations. First, a lack of liver biopsy data for classifying the severity of hepatic steatosis was evident. Second, this study only included only Uyghur populations, and results could not be extrapolated to other populations. Then, the overall study population was young, and the results could not be extrapolated to older populations. Finally, we did not intend to use these hepatic steatosis indexes to replace the gold standard or other clinical tests. These indicators were non-invasive and were more economical and convenient to obtain in large populations. The hope was to help a wider population recognize possible disease risks ahead of time in larger screenings.

## Conclusions

The FLI, HSI, ZJU, LAP, and VAI were associated with the risk of MAFLD in rural Uyghurs in Xinjiang. Among them, the FLI, HSI, ZJU, and LAP had good screening values for MAFLD. In conclusion, both the FLI and BMI could be used as simple screening tools for MAFLD in Uyghur populations of rural Xinjiang. These parameters may be used in large-scale epidemiological studies, which may help improve healthcare services in rural areas.

## Data Availability

The datasets used or analyzed during the current study are available from the corresponding author on reasonable request.

## Abbreviations

MAFLD: Metabolic dysfunction-associated fatty liver
FLI: Fatty liver index
ZJU: Zhejiang University Index
LAP: Lipid accumulation product
HIS: Hepatic steatosis index
VAI: Visceral adiposity index
ROC: Receiver operating characteristic
AUC: Areas under the receiver operating characteristic curve
BMI: Body mass index
WC: Waist circumference
HC: Hip circumference
SBP: Systolic blood pressure
DBP: Diastolic blood pressure
T2DM: Type 2 diabetes mellitus
FPG: Fasting plasma glucose
TG: Triglycerides
TC: Total cholesterol
HDL-C: High-density lipoprotein cholesterol
LDL-C: Low-density lipoprotein cholesterol
AST: Aspartate aminotransferase
ALT: Alanine aminotransferase
GGT: Gamma-glutamyl transferase
LR +: Positive likelihood ratio
LR-: Negative likelihood ratio
PPV: Positive predictive value
NPV: Negative predictive value
OR: Odds ratio
CI: Confidence interval
